# Estuaries as Filters: The Role of Tidal Marshes in Trace Metal Removal

**DOI:** 10.1371/journal.pone.0070381

**Published:** 2013-08-07

**Authors:** Johannes Teuchies, Wouter Vandenbruwaene, Roos Carpentier, Lieven Bervoets, Stijn Temmerman, Chen Wang, Tom Maris, Tom J. S. Cox, Alexander Van Braeckel, Patrick Meire

**Affiliations:** 1 Department of Biology, Ecosystem Management Research Group, University of Antwerp, Wilrijk, Belgium; 2 Flanders Hydraulics Research, Flemish Government, Antwerp, Belgium; 3 Department of Biology, Systemic Physiological and Ecotoxicological Research Group, University of Antwerp, Antwerp, Belgium; 4 Research Institute for Nature and Forest, Department of Biodiversity and Natural Environment, Brussels, Belgium; Plymouth University, United Kingdom

## Abstract

Flux calculations demonstrate that many estuaries are natural filters for trace metals. Yet, the underlying processes are poorly investigated. In the present study, it was hypothesized that intertidal marshes contribute significantly to the contaminant filter function of estuaries. Trace metal concentrations and sediment characteristics were measured along a transect from the subtidal, over an intertidal flat and marsh to a restored marsh with controlled reduced tide. Metal concentrations in the intertidal and restored marsh were found to be a factor two to five higher than values in the subtidal and intertidal flat sediments. High metal concentrations and high accretion rates indicate a high metal accumulation capacity of the intertidal marshes. Overbank sedimentation in the tidal marshes of the entire estuary was calculated to remove 25% to 50% of the riverine metal influx, even though marshes comprise less than 8% of the total surface of the estuary. In addition, the large-scale implementation of planned tidal marsh restoration projects was estimated to almost double the trace metal storage capacity of the present natural tidal marshes in the estuary.

## Introduction

When trace metals are discharged in aquatic systems they can be transported to the ocean, where management and remediation of contaminants become more difficult or impossible. Riverine input is calculated to be the major source of metals in the Greater North Sea [1] (OSPAR, Oslo/Paris convention for the Protection of the Marine Environment of the North-East Atlantic). Consequently, reduction of riverine metal fluxes is an important measure to protect marine ecosystems. First, the direct discharge of contaminants into the aquatic system has to be reduced by strict legislation and control. In addition, filtering processes by natural systems during the river-sea continuum can reduce the input of contaminants into the sea. Estuaries, which are transitional zones between terrestrial and marine waters, are found to be successful filters for contaminants [2], [3], [4], [5]. Mass balances of trace metals in the Schelde estuary, Belgium, SW Netherlands [6], [7], [8], [9] and other estuaries [5], [10], [11], [12] have been studied before. It was calculated that a large part of the trace metals accumulated in the high turbidity zone of the Schelde estuary [6]. However, estimations on the specific contribution of tidal marshes to this accumulation and the overall filter function of the Schelde or other estuaries with respect to trace metals are scarce.

In present study it is hypothesized that tidal marshes play an important role in the metal filter function of estuaries. When entering the estuary, trace metals are mainly associated with fluvial suspended matter (SPM) [13]. SPM and associated metals in estuaries accumulate mainly in areas with low hydrodynamic energy [14], [15], [16]. Tidal flooding of marshes results often in gradual accretion [17], [18], which is expected to be important for the removal of trace metals from the surface water in estuaries. These marshes are characterized by vegetation, sediments rich in litter and low flow velocities during flooding which promotes sedimentation of the fine grained fraction of suspended solids. This fraction is often rich in organic matter and clay particles and is known to display a high affinity for trace metals [13], [19]. Despite post-depositional diagenetic metal mobility and possible release to the surface water, the organic rich, hypoxic sediments of marshes are generally considered to be sinks for metals [20], [21], [22]. In a first part of present study, sediment characteristics and metal concentrations of surface sediments from a subtidal zone, an intertidal flat, an intertidal marsh and a restored marsh were investigated on one location in the freshwater stretch of the Schelde estuary, in order to evaluate differences in the filtering capacity of these areas. In a second part, the contribution of tidal marshes to the filter function of the estuary was calculated for the entire Schelde estuary.

The following hypotheses are tested:

Sediments deposited on tidal marshes are generally higher in clay, organic matter and trace metal content compared to subtidal and intertidal flat sediments.
Metal accumulation in tidal marshes contributes significantly to the contaminant filter function of estuaries.
The implementation of tidal marsh restoration projects, planned along the Schelde estuary, will increase metal removal by overbank sedimentation.

## Materials and Methods

### 1. Ethics statement

The field samples contained only sediments, so no endangered or protected species were involved. To sample these river sediments no specific permission was required.

### 2. Sampling

**Figure 1:**
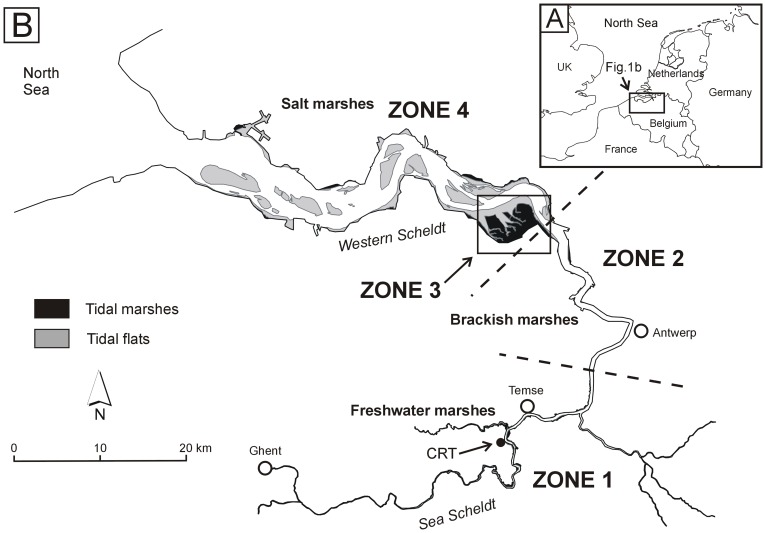
Map of the area. Location of the Schelde estuary (A) and the study area (CRT) within the estuary (B).

**Figure 2:**
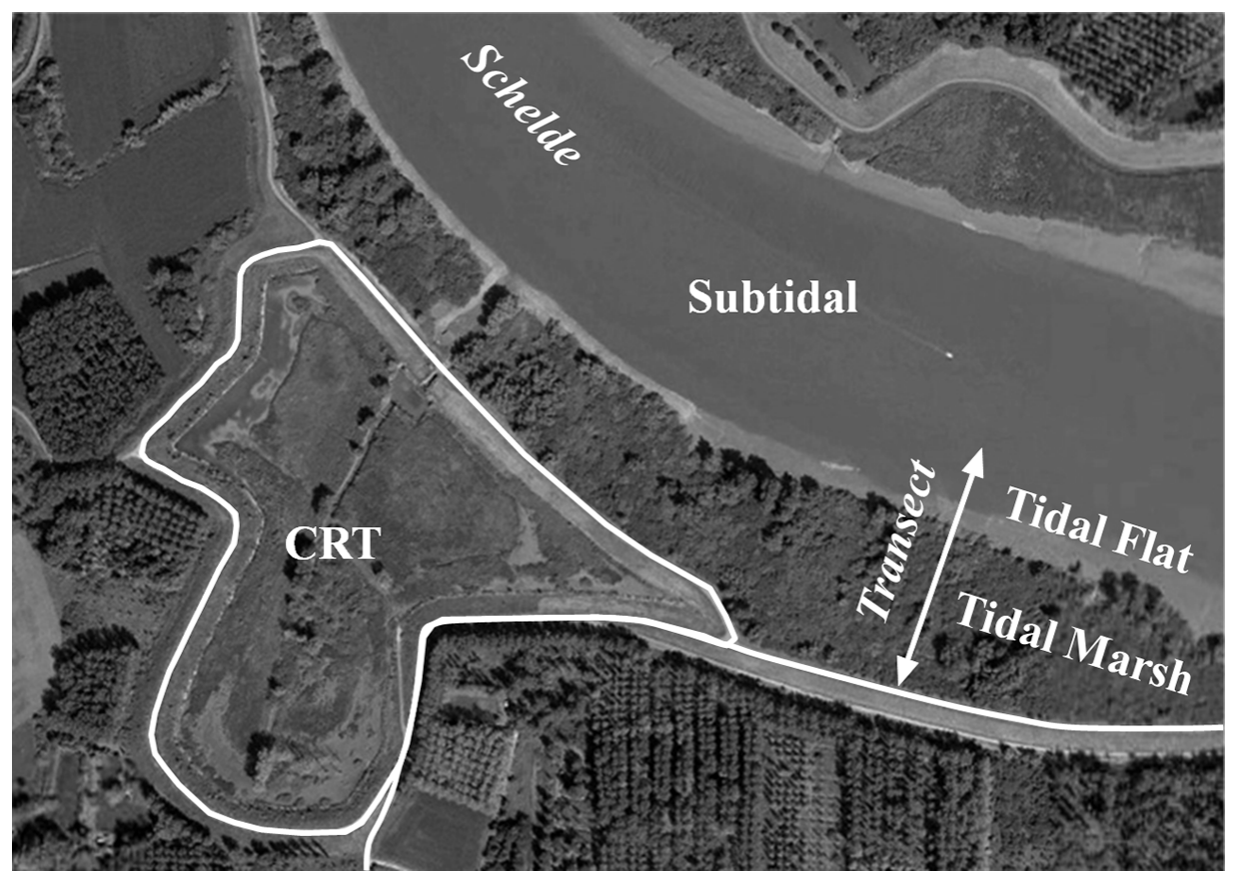
Detailed map of the study area. Sampling locations for the subtidal samples, tidal flat and tidal marsh samples along a transect and the location of the CRT.

The study is carried out in the Schelde estuary (Belgium and The Netherlands), a turbid, macrotidal and eutrophic system [23] with elevated metal concentrations in surface water and associated with suspended solids and sediments [24], [25], [26], [27]. For the present study, sediments were sampled along a transect from the subtidal, over a tidal flat and marsh to a restored marsh with controlled reduced tide (CRT) [28], [29], [30] all located in the freshwater tidal zone at 51°05′10′′N; 4°10′20′′E (Fig. 1, 2). Samples were taken in summer (August 2009, not subtidal) and winter (December 2009-January 2010). Subtidal sediment was sampled with a Reineck box corer (0–5 cm; 4 replicates within a distance of 10 m). On the tidal flat, one sediment sample (upper cm) was sampled with a plastic spoon every 5 m from the marsh edge to the water line (60 m in winter, 35 m in summer). In order to quantify sediment deposition rates, sediment samples were collected with sediment traps (PVC plates, diameter 20 cm) on the tidal marsh (13 locations evenly distributed on a transect from the marsh edge to the dike) and on the CRT (16 locations randomly spread over the area). Due to the high stream velocity above the tidal flat, no sediment traps could be used in this area. At every location, in the marsh and CRT, one trap with paper filter and one without filter were collected after 28 days exposure time (2 spring-neap tidal cycles). Comparable to the tidal flat sediments, the traps were exposed in summer (August 2009) and winter (December 2009, January 2010). Sediment traps with filter were used to collect and handle deposited sediments more easily: sediments were deposited on pre-dried and weighed filters. These filters with sediments were collected, oven dried (60 °C, until constant weight) and weighed. Sediments for analysis were collected from traps without filter. All sediment samples were transported cooled in polyethylene recipients from the field to the laboratory.

### 3. Analyses

For grain size analysis a subsample (1 g) of fresh sediment was boiled in 5 mL hydrogen peroxide (H_2_O_2_, 30%) to remove the organic matter. The clay (<2 µm) silt (2–63 µm) and sand (>63 µm) fraction of this mixture was determined with a laser diffraction particle size analyzer (Malvern S, Malvern Instruments Ltd, Worcestershire, UK). Organic matter (OM) was estimated through loss on ignition. For this purpose, the weight difference of oven dried (105°C) and incinerated sediment (after 6 h exposure at 550°C) was determined. Trace metal concentrations were determined in a mixture of an air dry subsample (0.2 g) with nitric acid (5 mL HNO_3_ 69%, Merck EMSURE^®^ for traces analysis) and hydrogen peroxide (5 mL H_2_O_2_ 30%, VWR Int. AnalaR NORMAPUR^®^ for traces analysis) after hot block digestion, 30 min at 100°C, cooled overnight and heated again for 60 min at 150°C. Metal concentrations were measured after filtration (cellulose mixed ester, 0.45 µm, Chromafil^®^) using an ICP-OES (inductively coupled plasma – optic emission spectroscopy, iCAP 6300 Duo SERIES Thermo Fisher Scientific®, Waltham, USA). Analytical accuracy was achieved by the use of blanks and certified reference material for sediment (Institute for Reference Materials and Measurements (IRMM), BCR^®^ № 320, channel sediment) included in each series of samples for metal analysis. Since the method used is not a total digestion, but extracts the *total recoverable metals*, the values from reference samples were lower but above 90% of the certified values for As, Cd and Zn, and above 80% for Cu, Ni, Pb and Mn. Recovery of Cr was low (50%). Variation between recoveries was very low for all elements.

### 4. Model calculations

A long-term marsh sedimentation model, MARSED, has been used to estimate the temporal variation in metal accumulation in a tidal marsh. Sediment and metal deposition rates have been simulated in a marsh which evolves from a young stage (i.e. low elevation and high flooding frequency) towards an older marsh with a higher elevation which is in equilibrium with the mean high water level (MHWL) and which consequently experiences a lower flooding frequency. The use and validation of this model is extensively described in Struyf et al. (2007) [31], Temmerman et al. (2003) [32] and Temmerman et al. (2004b) [15]. This model simulates sediment deposition rates based on the input of SPM concentrations and flooding frequency and duration, which depends on the tidal marsh height and river water level. The model is extended to simulate long term variation in metal deposition as a young, low marsh evolves to a high equilibrium marsh. As an example, the average Cd concentration in sediments from freshwater marshes of the Schelde estuary (7.19 µg g^−1^) calculated in this study (see below) is used as input for the model.

### 5. Budget calculations

In a second part of this manuscript the amount of trace metals which accumulated annually in tidal marshes was calculated for the entire Schelde estuary (tidally influenced tributaries not included due to insufficient data). The budget calculations are not an extrapolation of the results from the first part of present manuscript, but are based on a large dataset including personal data, data from governmental agencies and literature. The data includes surface areas of all tidal marshes (GIS data from governmental agencies), sediment accretion rates (personal data, data from governmental agencies and literature) and metal concentrations in tidal marshes and suspended solids (personal data including metal concentrations from the first part of this manuscript and governmental data) (Table S1, S2 for overview).

#### 5.1. Different zones in the estuary

The estuary was divided into four zones with similar characteristics in terms of metal concentrations and sedimentation. The first zone consists of freshwater marshes, which reach from km 160 in the estuary (Ghent) to km 85 (Fig. 1). This zone is most polluted since no dilution with marine water or sediments occurs. Zone 2 comprises all brackish marshes from km 85 up to the large brackish marsh of *Saeftinghe* (km 58, Dutch – Belgian border). Downstream of the border, the estuary becomes wider and the influence of tidally introduced marine water and sediments is much larger [33]. Due to its large area (2225 ha) the brackish marsh of *Saeftinghe* was considered as a distinct zone (zone 3). Zone 4 covers all brackish and salt marshes from *Saeftinghe* towards the mouth of the estuary (Westerschelde). Additionally, future metal accumulation was estimated in flood control areas with controlled reduced tide (CRT) and de-embankments projects which will be implemented along the fresh and brackish reach of the estuary.

The surface area of tidal marshes was based upon information of the vegetated tidal zones (GIS data from the *Flemish research institute for nature and forest* (INBO)). The surface area of the future CRT's and de-embankments is a total of operational areas (8 and 50 ha respectively), areas under construction (300 ha CRT) and areas approved by the Flemish government in 2010 (650 and 500 ha respectively) all as part of the Sigma plan (i.e. a project of the Flemish government which aims to reduce flood risk and at the same time will increase the ecological value of the Schelde estuary).

#### 5.2. Marsh accretion

Data on marsh accretion (m y^−1^) for zone 1 and 2 are based on the measurements from the INBO: marsh accretion between October 1996 and January 2000 was measured with respect to a white kaolin clay layer in the marsh soil on 15 freshwater marsh locations and on 16 brackish marsh locations (Table S1 for overview). Marsh accretion in zone 3 and 4 was calculated from changes in volume (i.e. surface area and elevation) of these areas based on digital elevation model (DEM) maps from 1963, 1992, 2002, 2004, 2009 and 2010 for *Saeftinghe* marsh (zone 3) and from 2004, 2009 and 2010 for zone 4. The DEMs for the periods before 2000 were computed from theodolite surveying with a minimum density of 1 point per 0.75 ha, and for the periods after 2000 based on LiDAR surveys with a minimum density of 1 point per 16 m^2^ (e.g., [15]). Sedimentation rates are in good agreement with other values reported in the Schelde estuary based on e.g. dating of sediment cores [15], [22].

Long term accretion in the CRT's is based on measurements and modeling of Vandenbruwaene et al. (2011) [18]. For the de-embanked areas the average of accretion in zone 1 and 2 was used. The bulk density value is based on measurements from Temmerman et al. (2004) [15]. Since no spatial trend in bulk density was observed along the estuary, an average bulk density of 494+/−122 kg m^−^^3^ (n = 17) was used for all zones.

#### 5.3. Metal concentrations

Trace metal values are based on concentration measurements from sediment traps and in superficial (0–10 cm) sediments sampled between 2000 and 2010 in 6 marshes (n = 120) for zone 1, in 2 marshes (n = 16) for zone 2 (personal data and data from INBO) (Table S2 for values). Values and trends in metal concentrations found in these zones are in accordance with available results from previous research [24], [25], [34], [35]. From *Saeftinghe* marsh onwards the estuary becomes wider and marine influence increases drastically (Fig. 1). In this zone 95% of the sediments are found to have a marine origin [33], [36]. Due to a lack of recent data on metal concentrations in marsh sediments from the Westerschelde (from border to mouth), trace metal values in zone 3 and 4 are based on concentrations in suspended solid sampled from 2005 to 2010 at 2 locations (km 58 and 35; n = 155) for *Saeftinghe* marsh (zone 3) and on 4 locations for zone 4 (km 35, 19, 0 and −10; n = 173) (data from *the Dutch ministry for infrastructure and environment, Rijkswaterstaat*). The values observed are within the range of metal concentrations in surface sediments from Westerschelde marshes (zone 4) reported by Beeftink et al. (1982) [37], lower (approximately half) of the concentrations reported in marshes by Zwolsman et al. (1993) [22] and slightly lower than suspended solid concentrations measured in 1995 [24]. This observation is in accordance with the expected decrease in sediment-bound metal concentrations due to a general improvement in water quality in recent decades [38], [39].

#### 5.4. Metal accumulation

For each zone total metal accumulation in the marshes *Am* (g y^−1^) is calculated:
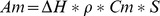
With Δ*H*  =  average annual accretion of the marsh (m y^−1^), *ρ*  =  average bulk density (10^3^ kg dw m^−3^), *Cm*  =  average metal concentration (g (10^3^ kg)^−1^ dw) and *S*  =  total surface of all marshes in the zone (m^2^). Values for *Am* are large and expressed as 10^3^ kg y^−1^.

The total metal accumulation of marshes in the Schelde estuary was compared with riverine metal input. Fluxes based on literature were found for the period 1981–1995. To compare with more recent fluxes, basic calculations for the period 2005-2010 were carried out: the average freshwater discharge (km 95, downstream from the Rupel, n = 84; 104±52 m^3^ s^−1^ was multiplied with the average total metal concentrations (km 85–110; n = 200; data from the *Flemish Environment Agency,* VMM). This is a rough calculation which probably underestimates the influx since metals deposited before this point and influx after this point are not taken into account. Yet, this calculated riverine input (e.g. 2.60 ton Cd y^−1^) is higher than the total influx (1.53 ton Cd y^−1^) from the three major tributaries (Bovenschelde, 0.73 ton Cd y^−1^; Dender 0.58 ton Cd y^−1^; Rupel 0.22 ton Cd y^−1^. The same method has been used previously for the Schelde [38].

### 5. Statistical analysis

The significance of differences between the sampled areas (subtidal – tidal flat – tidal marsh – CRT) in metal concentration and sediment or metal accumulation within a season was tested with a one way analysis of variance test (ANOVA). Differences between seasons within an area were tested with a paired t-test. Normality of the data was tested with the Shapiro-Wilk test prior to analysis. The Pearson correlation-coefficient was used to determine correlations between different metals and sediment characteristics over all areas and both seasons.

## Results

### 1. Spatial distribution and seasonal differences in trace metals in deposited sediments

**Figure 3:**
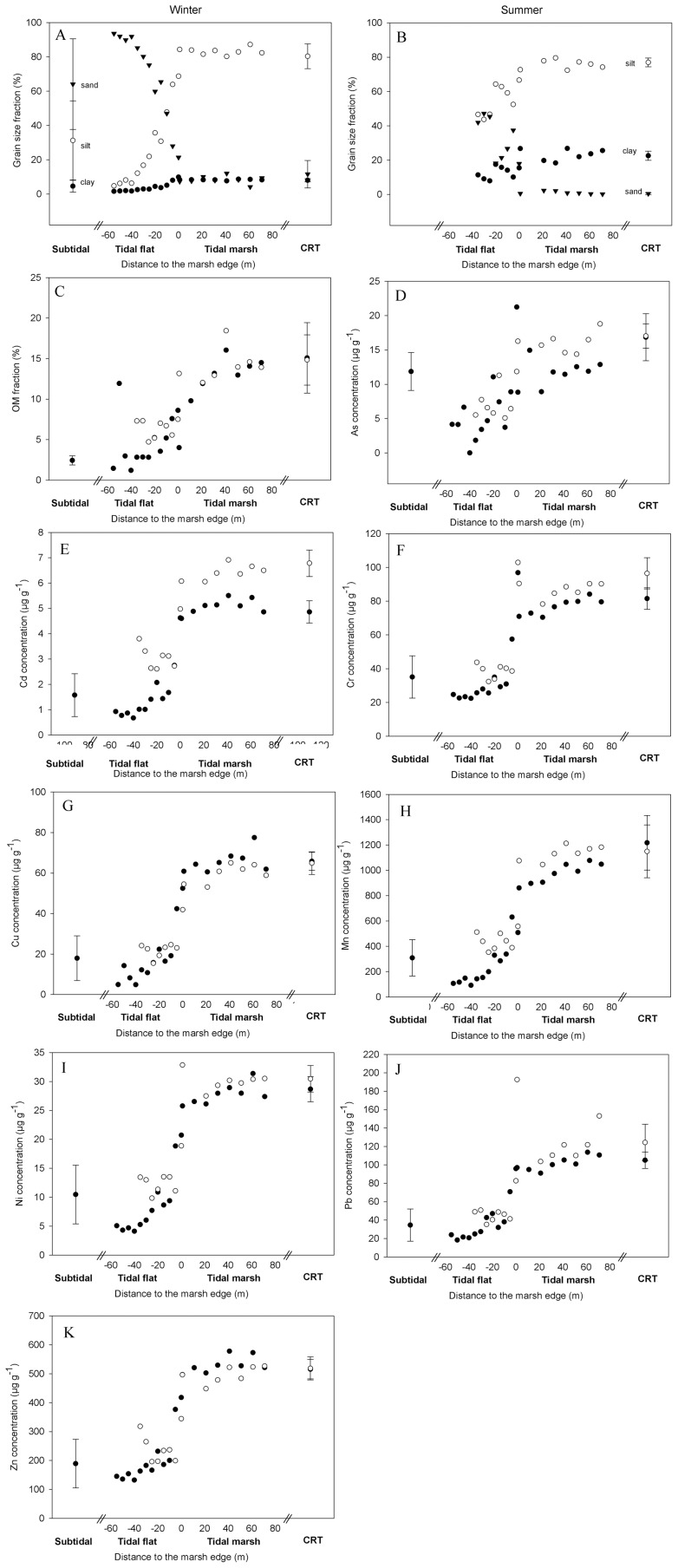
Grain size and metal concentrations in the different zones of the estuary. Grain size in winter (A), summer (B), organic matter (C) and metal concentrations (D–K) in estuarine sediments. For OM and metal concentrations: black for winter, white for summer. Average values with standard deviation for subtidal (n = 4, only winter) and restored marsh (CRT, n = 16) sediments.

In general, clay, silt, OM and metal concentrations were not significantly different between the subtidal and tidal flat sediments. In these subtidal and tidal flat sediments clay and silt content as well as metal concentrations were lower, they increased towards the marsh edge and were significantly higher (*p*<0.05, one way ANOVA) in the natural marsh and CRT in both seasons (Fig. 3, Table 1). Trace metal concentrations in the marsh and CRT were a factor 2–5 higher compared to concentrations in tidal flat sediments. For sand, the opposite trend was observed, with significantly (*p*<0.05) higher values in the tidal flat compared to the tidal marsh and CRT. Differences between the marsh and CRT were small and significantly different concentrations were only found for As, Cr and Cu (*p*<0.05). Spatial distribution was found to be similar for all metals studied (significant correlation, *p*<0.001, Table 2) and all metal concentrations were positively correlated with clay, silt and OM content and negatively with sand (*p*<0.001).

**Table 1:** Average metal concentrations (µg g^−1^) and sediment characteristics (% dw) in the different areas for winter and summer.

	Subtidal	Tidal Flat		Tidal Marsh		CRT	
	Winter	Winter	Summer	Winter	Summer	Winter	Summer
**As**	11.9^(abc)^	7.79^(a)^	7.54^(x)^	11.7(b)	15.0(y)	15.9^(c)^	17.0^(y)^
**Cd**	1.58^(a)^	2.00^(a)^	3.29^(x)^	4.90^(b)^	6.22^(y)^	4.76^(b)^	6.77^(y)^
**Cr**	35.1^(a)^	41.1^(a)^	46.6^(x)^	74.3(b)	83.7^(y)^	78.7^(b)^	97.0^(z)^
**Cu**	17.9^(a)^	23.9^(a)^	24.3^(x)^	63.2(b)	57.7^(y)^	63.8^(b)^	64.3^(z)^
**Mn**	308^(a)^	323^(a)^	447^(x)^	942(b)	1.10 103^(y)^	1.18 10^3(b)^	1.17 10^3(y)^
**Ni**	10.5^(a)^	10.9^(a)^	13.1^(x)^	26.9^(b)^	29.1^(y)^	27.8^(b)^	31.2^(y)^
**Pb**	34.6^(a)^	47.4^(a)^	49.4^(x)^	97.6^(b)^	121^(y)^	101^(b)^	135^(y)^
**Zn**	189^(a)^	241^(a)^	249^(x)^	515^(b)^	477^(y)^	500^(b)^	514^(y)^
**Clay**	4.62^(a)^	4.99^(a)^	12.7^(x)^	8.32^(b)^	23.3^(y)^	8.06^(b)^	22.6^(y)^
**Silt**	31.3^(a)^	37.2^(a)^	55.3^(x)^	82.2^(b)^	75.5^(y)^	80.3^(b)^	77.0^(y)^
**Sand**	64.1^(a)^	57.8^(a)^	32.0^(x)^	9.51^(b)^	1.26^(y)^	11.6^(b)^	0.46^(y)^
**OM**	2.44^(a)^	4.84^(a)^	6.42^(x)^	12.3^(b)^	14.0^(y)^	14.6^(b)^	15.2^(y)^

Significant differences (p<0.05) between seasons within an area are underlined and differences between areas within a season are indicated with letters (a, b, c for winter; x, y, z for summer).

**Table 2:** Correlation coefficients (R-values) between the different metals and sediment characteristics.

	Cd	Cr	Cu	Mn	Ni	Pb	Zn	clay	silt	sand	OM
**As**	0.762	0.814	0.768	0.762	0.79	0.693	0.759	0.518	0.578	−0.694	0.729
**Cd**		0.949	0.889	0.847	0.927	0.852	0.895	0.794	0.676	−0.906	0.805
**Cr**			0.921	0.859	0.937	0.868	0.919	0.679	0.71	−0.873	0.807
**Cu**				0.901	0.956	0.805	0.988	0.487	0.865	−0.893	0.843
**Mn**					0.959	0.845	0.906	0.575	0.728	−0.835	0.941
**Ni**						0.909	0.959	0.646	0.782	−0.911	0.904
**Pb**							0.814	0.724	0.585	−0.803	0.853
**Zn**								0.496	0.843	−0.881	0.848

All correlations were significant (p<0.001).

Grain size distribution and metal concentrations were different between sediments deposited in summer and winter (Fig. 2, Table 1). In general, sediments sampled during the summer campaign had a smaller grain size and higher metal concentration. These differences were most pronounced in the tidal marsh (all *p*<0.05, paired t-test) where clay, silt and metal concentrations were higher in summer, except for Cu and Zn concentrations which were higher in winter. Differences were large e.g. for clay with 8% dw in summer and 23% dw in winter and for Cd with 4.9 µg g^−1^ in winter and 6.2 µg g^−1^ in summer. Due to a smaller dataset and more spatial variation (gradient from the river to the marsh edge) these differences were less distinct in the tidal flat and only significant for the grain size parameters.

### 2. Metal accumulation in natural tidal marshes and CRT areas

**Figure 4:**
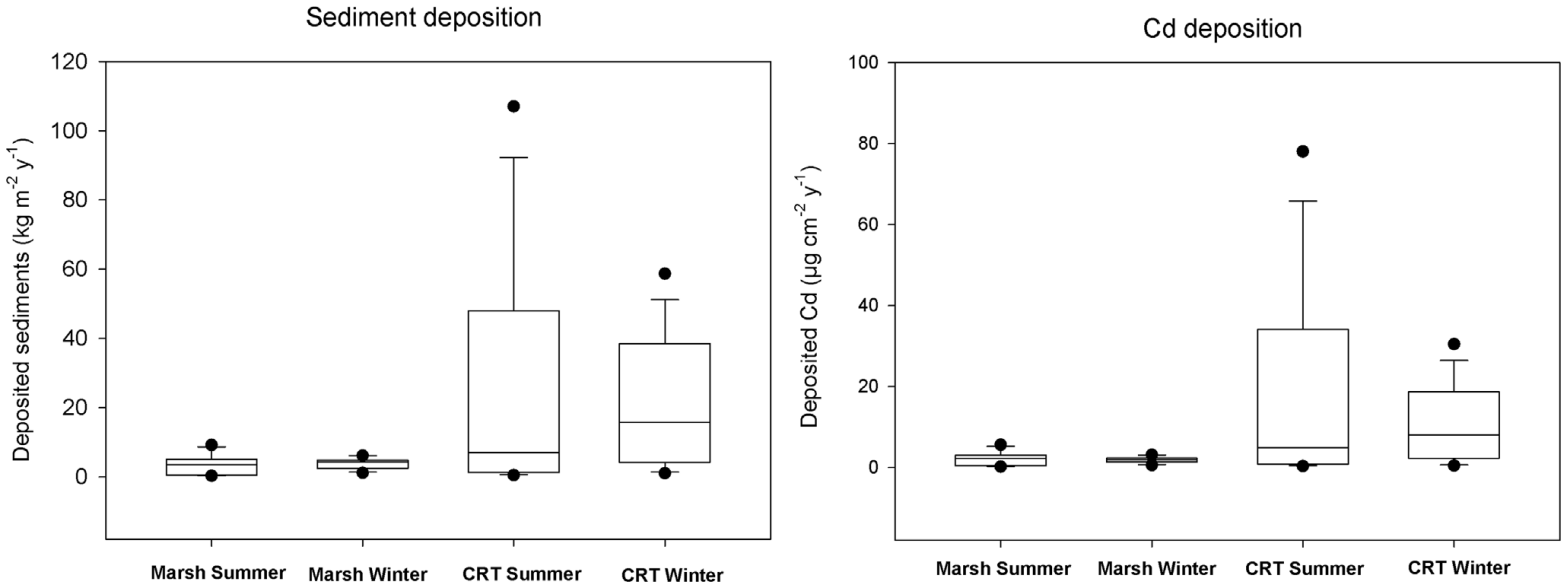
Sediment and metal deposition. Box plots (median, 25^th^, 75^th^ percentile and standard deviation) of seasonal difference in deposited sediments (A) and deposited Cd (B) in a tidal marsh and restored marsh (CRT).

The average sediment deposition rate, as measured with the sediment traps, was found to be higher (*p*<0.05, one way ANOVA) in the CRT (27±33 kg m^−^^2^ y^−1^ in summer; 22±18 kg m^−^^2^ y^−1^ in winter) than in the adjacent marsh (3.5±2.8 kg m^2^ y^−1^ in summer; 3.9±1.6 kg m^−^^2^ y^−1^ in winter) (Fig. 4). Variation between traps within the CRT was large. This spatial variation in sedimentation rates is mainly related to spatial variations in the marsh surface elevation: the lower the elevation, the higher the frequency, duration and depth of tidal inundations, which results in higher sedimentation rates.

The large sediment accumulation in the CRT coincided with a significant higher metal deposition compared to the marsh (*p*<0.05 for all metals in winter and summer, one way ANOVA). No significant differences (*p*>0.05, paired t-test) between the amount of accumulated sediments or metals were found between winter and summer in both areas.

**Figure 5:**
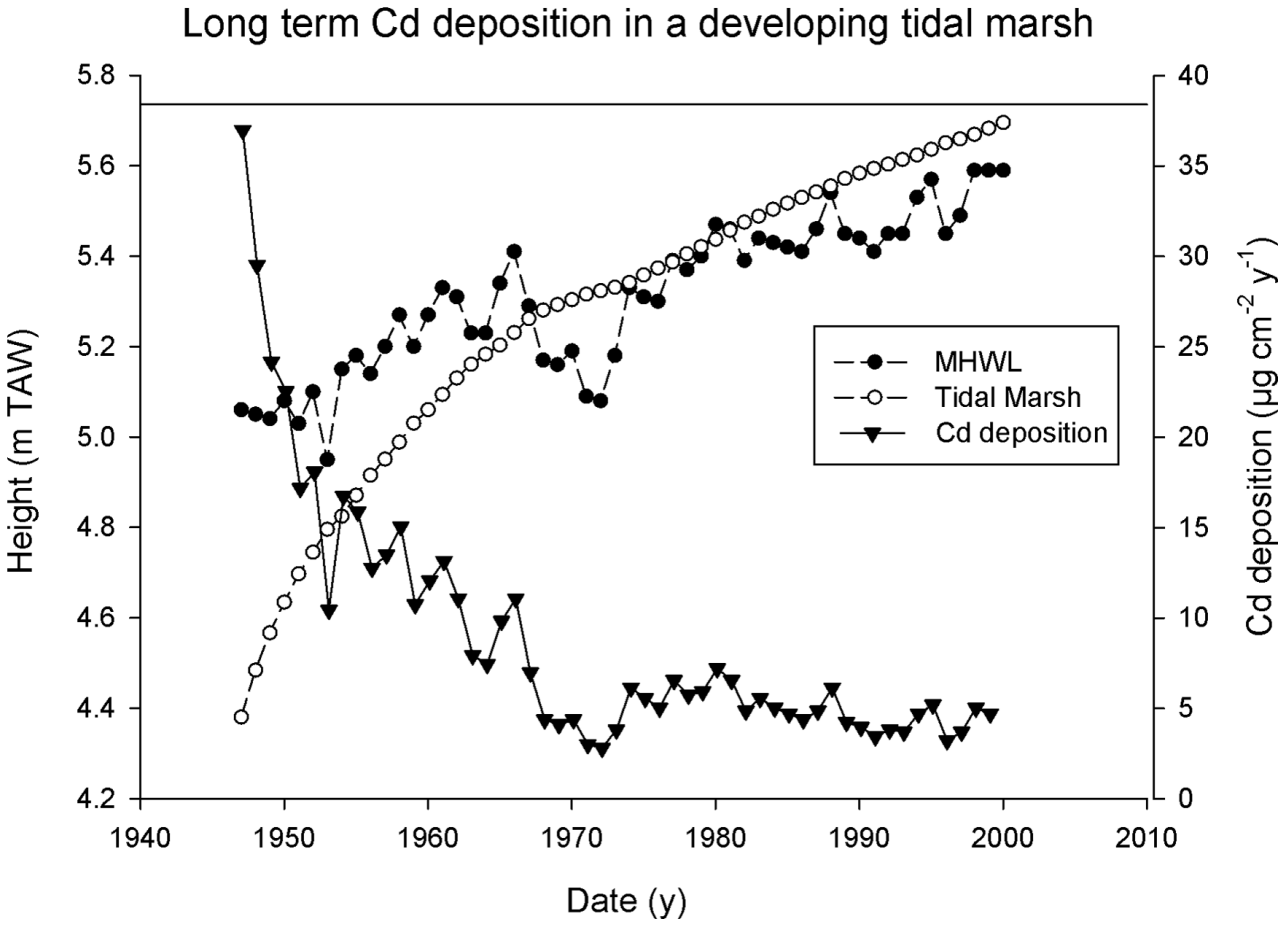
Modeled Cd deposition in a developping marsh. Modeled evolution of marsh elevation and Cd deposition. Elevation of the marsh and mean high water level (MHWL), both in m TAW (Belgian reference height) on the left Y-axis. Cd deposition (µg cm^−2^ a^−1^) on the right Y-axis.

The effect of marsh surface elevation on sediment and metal accumulation is further illustrated by modeling the temporal variation in metal accumulation of a young, low elevated marsh developing towards its high elevated equilibrium state (Fig. 5). Average freshwater tidal marsh Cd concentrations (see 2.2 and Table S1) were used as an example. Low marsh elevation resulted in high sedimentation rates and high Cd accumulation (almost 40 µg cm^−2^ y^−1^). With increasing marsh elevation, tidal inundation frequency, duration and depth diminished, so that sediment and Cd accumulation rapidly decreased and became constant (around 5 µg Cd cm^−2^ y^−1^) after 20 years, when marsh elevation attained an equilibrium with the mean high water level.

Average metal concentrations in surface sediments of tidal marshes were estimated for the different estuarine zones (see 2.2.). A general trend existed with the highest metal concentrations in the freshwater marshes and decreasing values towards the mouth of the estuary (Table 3), although no clear trend was apparent for As, Cr, Mn or Ni. Average sedimentation rates were comparable in the different zones (6.5–9.3 kg m^2^ y^−1^) but were approximately double (16.3 kg m^2^ y^−1^) in the CRT areas. This resulted in the highest metal deposition rates per surface unit in the CRT's. In the different zones, the rate of metal deposition was highest in the freshwater marshes with the exception of As. Due to the high estimated sedimentation rates and the significant surface area (950 ha) planned to be implemented, the total metal retention by future CRT's is estimated to be comparable to the metal accumulation in all present marshes in the Schelde estuary. For the different zones of the existing marshes, metal accumulation is highest in *Saeftinghe* marsh, which can be attributed to its large areal extent (2225 ha).

**Table 3:** Average metal concentrations (µg g^−1^), calculated metal deposition per surface unit (µg cm^−2^ y^−1^) and estimated total accumulated metals in the different zones of the Schelde estuary (10^3^ kg y^−1^).

		Zone 1	Zone 2	Zone 3	Zone 4		
		Freshwater marshes	Brackish marshes	*Saeftinghe *marsh	Brackish/Salt marshes	Future CRT's	Future marshes
Concentration (µg g^−1^)	As	17.8	23.7	22.5	17.9	20.8	20.8
	Cd	7.19	3.53	2.77	0.779	5.36	5.36
	Cr	110	63.5	99.2	81.7	86.9	86.9
	Cu	94.6	72.4	50.2	25.6	83.5	83.5
	Mn	1077	835	1285	884	956	956
	Ni	33.7	23.7	29.1	24.7	28.7	28.7
	Pb	133	80.2	76.4	48.0	106	106
	Zn	788	350	300	147	569	569
Sedimentation (kg m^−2^ y^−1^)		**9.36**	**8.07**	**6.48**	**7.52**	**16.3**	**8.71**
Metal deposition (µg cm^−2^ y^−1^)	As	16.7	19.1	14.6	13.5	33.8	18.1
	Cd	6.73	2.85	1.79	0.585	8.74	4.67
	Cr	103	51.2	64.2	61.4	142	75.8
	Cu	88.6	58.4	32.5	19.2	136	72.8
	Mn	1008	673	832	664	1558	833
	Ni	31.6	19.1	18.9	18.6	46.8	25.0
	Pb	124	64.7	49.5	36.1	174	92.8
	Zn	737	283	195	111	928	496
Surface (ha)		**262**	**153**	**2225**	**619**	**950**	**550**
Metal deposition (ton y^−1^)	As	0.436	0.293	3.24	0.834	2.64	0.995
	Cd	0.176	0.0436	0.399	0.0362	0.68	0.257
	Cr	2.71	0.783	14.3	3.80	11.1	4.17
	Cu	2.32	0.893	7.24	1.19	10.6	4.00
	Mn	26.4	10.3	185	41.1	122	45.8
	Ni	0.826	0.292	4.19	1.15	3.65	1.38
	Pb	3.25	0.989	11.0	2.23	13.5	5.10
	Zn	19.31	4.32	43.3	6.85	72	27.3

The average estimated sedimentation rate (kg m^−2^ y^−1^) and total surface for the marshes (in 2012) and expected future areas displayed.

Metal deposition in the marshes of the Schelde removes a considerable amount of trace metals from the surface water (23%–53% of the riverine input) (Table 4). With the implementation of marsh restoration projects as foreseen in the next decades, this fraction will roughly double (39%–90%).

**Table 4:** Estimated input and output of trace metals in the Schelde estuary (10^3^ kg y^−1^), based on literature and own calculations (^a^Baeyens et al. (1997) [6]; ^b^De Smedt et al. (1997) [8]; ^c^Baeyens et al. (2005) [38]; ^d^This study).

in 10^3^ kg per year	Period	As	Cd	Cr	Cu	Mn	Ni	Pb	Zn
**Freshwater input**	1981–1983^a^	-	12.36	-	123.6	-	-	125	386
	1990^b^	-	-	99.3	62.5	-	12.9	120	349
	1995–1998^c^	-	3.86	-	51.2	-	33.0	94.0	283
	2005–2010^d^	22.5	2.60	41.1	40.0	723	23.8	50.9	253
**Marine output**	1981–1983^a^		0.770		11.1			14.4	42.4
	1995–1998^c^		0.271		6.66		8.2	6.99	25.3
**Annual metal deposition marshes**	2005–2010^d^	5.06	0.655	21.6	11.6	271	6.46	17.5	73.8
	future^d^	8.70	1.59	36.8	26.3	438	11.5	36.1	173
**% removal by marshes**	2005–2010^d^	23	25	53	29	38	27	34	29
	future^d^	39	61	90	66	61	48	71	68

Total metal deposition (tons per year) and metal removal in marshes of the entire estuary compared the estimated riverine input (%), for the period 2005–2010 and for a future scenario with an additional surface area of restored marshes implemented.

## Discussion

### 1. Trace metal accumulation in a freshwater tidal area

Due to large-scale embankments and dredging, the Schelde estuary often has a deep and narrow streambed channel and large highly dynamic areas characterized by high flow velocities and high physical stress [23]. In these zones sedimentation of larger particles together with erosion of fine material occurs, which explains the very high sand (>63 µm) content in the investigated tidal flat and subtidal sediments (Fig. 3A). Frequent sedimentation measurements on this tidal flat demonstrated irregular sedimentation-erosion cycles (Beauchard, unpublished data) in contrast to constant accretion observed in most tidal marshes [17]. The coarse fraction (>32 µm) of Schelde sediment was found to contain mainly quartz, calcite and feldspar which are minerals with a low affinity for trace metals [13] explaining the lower metal concentrations in the investigated subtidal and tidal flat sediments (Fig. 3).

Trace metal concentrations in sediments deposited on the tidal marsh and CRT were a factor two to five times higher compared to the tidal flat (Fig. 3). Lower flow velocity in these areas, due to lower water depths and hydraulic friction by the marsh vegetation [40], resulted in deposition of the fine-grained sediment fraction. This fraction consisted mainly of clay, silt and organic matter and is known to have a high affinity for trace metals: clay particles can bind trace metals directly due to charged surfaces and act, as other small particles, as high-surface-area carriers which are coated with interlayered mixtures of organic matter and Fe and Mn oxides which form a solid surface with a strong affinity for metals [41]. The pattern for As is slightly different from the other metals with higher concentrations in subtidal sediments. Yet, this metalloid is also found to be associated mainly with Fe and Mn (hydr)oxides, organic matter and clay particles [42].

Sediments deposited in winter had a larger grain size and lower metal concentrations for most elements (Fig. 3 and Table 1). Lower winter metal concentrations in surface sediments of an urban estuary can be explained by resuspension of fine sediments caused by higher turbulence in winter [43]. Also in the Schelde estuary a strong seasonal pattern is observed in riverine and overmarsh SPM concentrations and sediment deposition rates on marshes, with higher values in winter. However no differences in grain size were reported in these studies [31], [44], [45]. No difference in sediment quantity between summer and winter was observed in present study, which may be because seasonal differences were only based on one spring-neap tidal cycle for both seasons.

As in other studies, trace metals were found to be mainly associated with the fine grained, organic rich fraction of the suspended solids (Table 2) [19]. When sediments are transported to the sea, a substantial part of this fine fraction and associated metals can accumulate within the estuary [36]. Based on the constant accretion and the fine and organic characteristics of the deposited sediments in the marsh and CRT of present study, these areas are expected to be important for the contaminant filter function of estuaries.

### 2. Trace metal accumulation in marshes of the entire estuary

Annual metal deposition per marsh surface area in the Schelde estuary varied considerably between the investigated zones (Table 3). Metal accumulation is highest in the CRT's, mainly due to their high sedimentation rates. The CRT is characterized by a stagnant phase during flood and a large spatial variation in elevation [28] which resulted in deposition of 24±26 kg dw sediment m^−2^ y^−1^ (Fig. 4). Elevation differences are expected to decrease in time and the CRT will progressively evolve towards a flattened platform with sedimentation rates of approximately 16 kg dw sediment m^−2^ y^−1^ [18], which is still double the estimated sedimentation rates in other marshes in the estuary. However, differences in locations in the estuary, initial topography or inlet/outlet configurations can result in other sediment accretion rates in future CRT areas.

Variation in sediment deposition on tidal marshes is strongly related to tidal inundation characteristics which, in turn are determined mainly by marsh elevation [45]. Our modeled results demonstrated that fast accretion rates occur in young, low elevation marshes (Fig. 5). The rapid rise of the marsh elevation results in lower inundation frequency, duration and depth with lower accretion rates as a consequence. In the long term (after about 2 to 3 decades) accretion rates in the marshes of the Schelde are found to be in equilibrium with the mean high water level rise in the estuary [15]. The decrease in sedimentation rates during marsh development coincided with an eightfold decrease in Cd deposition after twenty years. In the existing natural marshes metal accumulation per surface area is largest in the freshwater region, while differences between brackish marshes, *Saeftinghe* marsh or salt marshes are smaller (Table 3). The calculated Cd deposition per surface area in the freshwater marshes (zone 1; 6.3 µg cm^−2^ y^−1^) was more than 10 times higher compared to deposition in salt marshes (zone 4; 0.59 µg cm^−2^ y^−1^). These differences can be attributed to variation in the load, characteristics and metal concentrations of SPM and to differences in sedimentation rates which exist along the estuarine gradient. Riverine SPM and associated metals undergo changes due to mixing with marine water and sediments [14]. A decrease in metal concentrations in SPM observed towards the mouth is mainly due to dilution of contaminated riverine particles with less contaminated marine particles [14]. This is also observed in the estimated concentrations in the marshes of the different zones of the Schelde estuary for most elements (Table 3). However, this pattern obtained by conservative mixing can be changed by modifications in the distribution coefficient K_D_ (ratio of particulate versus dissolved trace metals) due to environmental variables in the surface water such as salinity, pH or redox potential [46]. An increase in dissolved Cd, Cu and Zn is often observed with the influence of marine water: The hypoxic conditions in the upper estuary promote the precipitation of dissolved Cd, Cu and Zn with sulfides while the dissolution of these complexes occurs under the oxic conditions of marine water [47]. Additionally, the formation of metal-chloride complexes increases the mobility of mainly Cd and Zn [47], [48]. Dissolved Cu is mainly associated with organic ligands which are progressively mineralized during transport to the sea, resulting in higher dissolved concentrations [14]. Dissolved Mn concentrations display an opposite trend, with lower concentrations in the lower estuary due to precipitation as oxy-hydroxides under the oxic conditions of marine water [47]. Also As and to a lesser degree Cr and Ni concentrations are found to behave similarly to Mn which can be attributed to association of these elements with Fe and Mn oxy-hydroxides under oxic conditions [7], [49]. Baeyens et al. (1997) [50] found that Cr, Ni and Pb exhibited a conservative mixing behavior in the Schelde estuary. These trends from literature are in good agreement with the difference in trace metal concentrations estimated in the marshes of the different zones of the Schelde estuary (Table 3). Due to the stagnant phase during high tide in CRT's, deposition of a finer fraction with higher metal concentrations can be expected. However, almost no differences in grain size, OM or metal concentrations between the CRT and the adjacent marsh were observed (Table 1).

### 3. Trace metal fluxes in the estuary

The calculated metal accumulation per unit surface area is found to be higher in the Schelde marshes compared to other marshes as summarized by Rozan and Benoit (1999) [51]. This can be attributed to the relatively high metal concentrations in SPM and high sedimentation rates in the Schelde estuary. However, the total metal accumulation in the marshes of an estuary depends largely on the total surface of the marshes. Less than 8% of the total surface area of the Schelde estuary consists of tidal marshes, 3300 ha, of which the brackish marsh *Saeftinghe* comprises 60% (Table 3). In order to protect the hinterland against flooding and to restore estuarine habitats, a large surface (950 ha) of flood control areas with controlled reduced tide (CRT) and marsh restoration projects (550 ha) are under construction or planned, mainly in the fresh- and brackish water part of the Schelde estuary (Sigmaplan 2012: personal communication). For the entire estuary an annual estimated amount of 0.7 ton Cd, 18 ton Pb and 74 ton Zn are deposited in the marshes (Table 4). In a future scenario, after implementation of an additional surface of 1500 ha of intertidal areas, metal accumulation in the Schelde estuary is calculated to be 1.6 (e.g. Mn) up to 2.3 times higher (e.g. Cd, 1.6 ton). Riverine Cd input was calculated to be 2.6 ton per year. The assessed estuarine metal removal by deposition in tidal marshes ranged from 23% (As) up to 53% (Cr) and increases up to 39% (As) –90% (Cr) with the implementation of 1500 ha of marsh restoration projects. However, it must be noted that extrapolation of the results from the small CRT to the planned large scale restoration projects may not be completely correct since implementation of these areas may change the sediment and metal balance of the estuary.

Mass balances of trace metal transport through the Schelde estuary have been calculated before and are closely linked to sediment fluxes. SPM fluxes originating from the river basin are mainly deposited in the upper estuary and SPM concentrations downstream of *Saeftinghe* marsh consist of almost 90% marine material [36]. A large fraction of fluvial SPM is found to be deposited in the high turbidity zone of the estuary (between Antwerp and the border, zone 2) [33]. Bottom sediments are found to be rich in silt and clay in this zone, while outside this stretch, bottom sediments consist mainly of sand, as observed in the investigated subtidal and tidal flat sediments of present study [52]. The yearly riverine input of sediments is variable and estimated to be on average 214±99 10^6^ kg for the period 1992–2007 [53], 430 10^6^ kg by Baeyens et al. (1997) [50] and 800 10^6^ kg by Sas and De Jonghe (1993) [54]. Our calculations on sediment accumulation were 230 10^6^ kg per year in present marshes and 470 10^6^ kg per year after the implementation of marsh restoration projects. Since these values comprise accumulation of marine and fluvial sediments, comparison is difficult. Yet, we can conclude that sedimentation in marshes may have an important effect on sediment balances in the estuary.

In a comparable way as for SPM, the estuary acts as a sink for trace metals and only a small fraction of the riverine input reaches the sea [9], [38]. The results of different studies estimating metal fluxes through the Schelde estuary are very similar [8], [24], [38], [55], [56]. The total riverine Cd input is estimated at 12 tons per year in 1981–1983 and 3.9 ton per year in 1995–1998 while the marine output remained low, around 0.8 and 0.3 ton per year respectively (Table 4). Metal input estimations decrease with time, determined by the general decline in metal pollution from 1960 to recent years [22], [38]. Linked to the SPM fluxes, Baeyens et al. (2005) [38] calculated that about 60% of the particulate metal phase is deposited in the maximum turbidity zone, while the marine output flux ranges from 7% (Cd) to 24% (Ni) of the input flux. These studies indicate that up to 90% of the trace metals are removed from the surface water in the Schelde estuary. Based on the calculations of present study, accumulation by tidal marshes contributes about 25%–50% to this filtering function of this estuary while they comprise less than 8% of the total surface of the estuary. Sedimentation in subtidal zones and dredging with terrestrial disposal are other main sinks for contaminants associated with SPM [9]. In order to ensure access for larger container ships to Antwerp harbor, deepening of the navigation channel and maintenance dredging is large and dredged volumes increased from ±7 10^6^ m^3^ fresh material y^−1^ between 1950 and 1970 up to an average of ±15 10^6^ m^3^ fresh material y^−1^ recent years (Flemish government). However, most of the dredged material is relocated in the estuary, and only a small part (e.g. in 2007, ±3%, i.e. 300 10^3^ m^3^ fresh material) is disposed on land [57].

### 4. Uncertainties of budget calculations

The flux calculations executed in this study are based on best available data. Certain processes and assumptions which may have had an effect on the results are considered. Trace metal concentrations in SPM or superficial sediment layers are used to calculate metal accumulation in marshes. However, a difference in SPM and deposited sediments may exist and processes as atmospheric deposition and postdepositional diagenetic mobility can change metal concentrations in superficial marsh sediments [22]. Nevertheless, metal concentrations in the 4 different zones are in agreement with values reported in literature from marshes and SPM [22], [25], [37], [38] with a spatial trend for lower concentrations towards the mouth as expected and described in 4.2. [14].

The uncertainties of metal budget estimates are usually hard to assess [5]. The variables used to calculate fluxes display a certain variability which resulted in a large uncertainty on the final flux values. Trace metal concentrations in SPM or marsh sediments had an average relative error (based on the standard deviation) of 30%. Relative errors of accretion rates and bulk density were 40% and 25% respectively. Additionally, freshwater discharge and water metal concentrations, used to calculate riverine metal input, both had a relative error of 50%. Calculation of metal removal by marshes and propagation of the uncertainties resulted in a large relative error of 180%. This emphasizes that given fluxes are not exact numbers. Yet, values used for the calculations and estimated fluxes were plausible and in accordance with the literature.

### 5. Estuaries as filters for trace metals

The Schelde estuary is found to be an effective filter for trace metals, reducing the metal input into the marine environment. Total metal fluxes in the North Sea were calculated within the framework of the OSPAR Commission, a treatise on ‘protecting and conserving the North-East Atlantic and it resources' [1]. A total riverine Cd input of 50 tons in 1990 and 20 tons in 2006 was estimated for the Greater North Sea (OSPAR region II). Additionally, 23 and 13 tons were deposited atmospherically in this region in 1990 and 2006 respectively. With an estimated Cd flux of 0.3 ton to the North Sea in 1995 (Table 4), the Schelde estuary contributes only for a very small fraction to metal fluxes in this region. The catchment areas from the Elbe and Maas/Rhine, which are much larger, have an annual influx of respectively 3 and 4 tons Cd in to the North Sea (2000–2006).

Different studies demonstrate the filtering capacity of estuaries with respect to trace metals. Large metal retention was observed in the Humber estuary, UK (10–97%) [3], [11] and in the Morlaix River estuary, France (86% for Pb) [5]. In the Gironde estuary (France), metal removal was small, and the estuary acted as a source for dissolved metals as Cd and Cu [10]. Also in the Seine river basin (France), retention was small (0.2 ton Cd) compared to the inflow (3.4 tons Cd) [12]. In last study, a small fraction (0.8%) of the retention was attributed to metal accumulation in floodplain soils of this river system [12]. Greater metal removal (19% Cu –45% Zn) by overbank sedimentation in the well developed floodplains of the river Swale (UK), contaminated by historical mining activities have been reported [58]. A salt marsh in the Quinnipiac estuary (USA) retained 6% (Cd) up to 17% (Pb) of the riverine metal input [51] and Rozan and Benoit (2001) [59] estimated a metal removal of 20%–30% of the riverine input by salt marshes in the same region. Despite the low surface area of tidal marshes in the Schelde estuary, comparable values for metal retention by marshes (23%–53%) were estimated (Table 3). High turbidity and increasing MHWL in the Schelde estuary might have caused high annual sediment accretion rates and hence metal accumulation per surface unit in Schelde marshes (Table 2) compared to values reported [51]. In the present study, the implementation of 1500 ha of intertidal areas was calculated to roughly double metal accumulation in the estuary, with an extra storage of about 100 ton Zn y^−1^ or 20 ton Pb y^−1^. Andrews et al. (2008) [2] calculated an extra storage of 6 ton Zn y^−1^ and 3 ton Pb y^−1^ as a result of a managed realignment scenario in which 26000 ha of intertidal areas will be restored in the Humber estuary (UK). These values are much lower, which can be attributed to the lower metal concentrations and lower accretion rates (1 mm y^−1^) used [2]. Also Andrews et al. (2006) [4] calculated an increase up to 100% of the current annual storage of trace metals in the Humber estuary (UK) in a large realignment scenario. Comparable to the present study, it was concluded that the storage of contaminants in the realignment sites enlarges the natural trace metal storage capacity of the estuary, which can improve the long-term water quality and avoid cleaning costs [3], [4].

The filter function of estuaries reduces contamination of the marine ecosystem, but results on the other hand in accumulation of pollutants and severe contamination of the estuarine habitat. Estuaries from all over the world are found to be contaminated [60], [61], [62], [63] which can result in toxicity and adverse effects on the biota living in the estuary [64].

## Conclusions

The results of this study indicate that overbank sedimentation in tidal marshes removes a substantial fraction (about 30%) of the riverine metal influx in the Schelde estuary, even though marshes comprise less than 8% of the total surface of the estuary. Metal accumulation is largest in freshwater marshes since dilution with less contaminated marine water resulted in lower metal concentrations in deposited sediments of brackish and salt marshes. Consequently, freshwater marshes have a higher filtering potential and accordingly are often most contaminated. The implementation of 1500 ha flood control areas and tidal marsh restoration projects planned along the Schelde estuary was calculated to almost double the metal accumulation capacity by overbank sedimentation, mainly caused by their high accretion rates. However, high uncertainties indicate that calculated values should be considered as order of magnitude estimates.

Fluxes of trace metals were found to be closely related to transport of suspended solids. The constant accretion in Schelde marshes, caused by the MHWL rise, determines to a large extent the metal accumulation capacity of the marshes. Sedimentation in subtidal areas of the maximum turbidity zone, with land disposal after dredging, is expected to be another important sink for sediments and associated contaminants in the Schelde estuary.

## Supporting Information

Table S1 **Overview of the data used for the calculation of the sediment deposition on the marshes in the different zones of the estuary.**
(DOCX)

Table S2 **Metal concentrations in sediments (from cores or sediment traps) used for the calculation of metal deposition in the Schelde estuary.**
(DOCX)

## References

[1] OSPAR (2009) Trends in waterborne inputs. Assessment of riverine inputs and direct discharges of nutrients and selected hazardous substances of nutrients and selected hazardous substances to OSPAR maritime area in 1990–2006.

[2] Andrews JE, Samways G, Shimmield GB (2008) Historical storage budgets of organic carbon, nutrient and contaminant elements in saltmarsh sediments: Biogeochemical context for managed realignment, Humber Estuary, UK. Sci Total Environ 405: 1–13. 18762324 10.1016/j.scitotenv.2008.07.044

[3] Cave RR, Andrews JE, Jickells T, Coombes EG (2005) A review of sediment contamination by trace metals in the Humber catchment and estuary, and the implications for future estuary water quality. Estuar Coast Shelf Sci 62: 547–557.

[4] Andrews JE, Burgess D, Cave RR, Coombes EG, Jickells TD, et al (2006) Biogeochemical value of managed realignment, Humber estuary, UK. Sci Total Environ 371: 19–30. 16996577 10.1016/j.scitotenv.2006.08.021

[5] Monbet P (2006) Mass balance of lead through a small macrotidal estuary: The Morlaix River estuary (Brittany, France). Mar Chem 98: 59–80.

[6] Baeyens W, Elskens M, Van Ryssen R, Leermakers M (1997) The impact of the Scheldt input on the trace metal distribution in the Belgian coastal area (results of 1981–1983 and 1995–1996). Hydrobiol 366: 91–108.

[7] De Gieter M, Elskens M, Baeyens W (2005) Fluxes and major transport routes of Arsenic in the Scheldt estuary. Mar Chem 95: 15–30.

[8] De Smedt F, Vuksanovic V, Van Meerbeeck S, Reyns D (1997) A Time-dependent flow model for heavy metals in the scheldt estuary. Hydrobiol 366: 143–155.

[9] Ouboter M, Van Eck B, Van Gils J, Sweerts J, Villars M (1997) Water quality modelling of the western Scheldt estuary. Hydrobiol 366: 129–142.

[10] Audry S, Blanc G, Schäfer J, Guérin F, Masson M, et al (2007) Budgets of Mn, Cd and Cu in the macrotidal Gironde estuary (SW France). Mar Chem 107: 433–448.

[11] Millward GE, Glegg GA (1997) Fluxes and retention of trace metals in the Humber Estuary. Estuar Coast Shelf Sci 44 Supplement 197–105.

[12] Thévenot DR, Moilleron R, Lestel L, Gromaire M-C, Rocher V, et al (2007) Critical budget of metal sources and pathways in the Seine River basin (1994–2003) for Cd, Cr, Cu, Hg, Ni, Pb and Zn. Sci Total Environ 375: 180–203. 17267024 10.1016/j.scitotenv.2006.12.008

[13] Bouezmarni M, Wollast R (2005) Geochemical composition of sediments in the Scheldt estuary with emphasis on trace metals. Hydrobiol 540: 155–168.

[14] Regnier P, Wollast R (1993) DISTRIBUTION OF TRACE-METALS IN SUSPENDED MATTER OF THE SCHELDT ESTUARY. Mar Chem 43: 3–19.

[15] Temmerman S, Govers G, Wartel S, Meire P (2004) Modelling estuarine variations in tidal marsh sedimentation: response to changing sea level and suspended sediment concentrations. Marine Geology 212: 1–19.

[16] Van Damme S, Frank D, Micky T, Olivier B, Eric S, et al (2009) Tidal exchange between a freshwater tidal marsh and an impacted estuary: the Scheldt estuary, Belgium. Estuar Coast Shelf Sci 85: 197–207.

[17] Temmerman S, Govers G, Meire P, Wartel S (2004) Simulating the long-term development of levee–basin topography on tidal marshes. Geomorphology 63: 39–55.

[18] Vandenbruwaene W, Maris T, Cox TJS, Cahoon DR, Meire P, et al (2011) Sedimentation and response to sea-level rise of a restored marsh with reduced tidal exchange: Comparison with a natural tidal marsh. Geomorphology 130: 115–126.

[19] Du Laing G, Rinklebe J, Vandecasteele B, Meers E, Tack FMG (2009) Trace metal behaviour in estuarine and riverine floodplain soils and sediments: A review. Sci Total Environ 407: 3972–3985. 18786698 10.1016/j.scitotenv.2008.07.025

[20] Spencer KL, Cundy AB, Croudace IW (2003) Heavy metal distribution and early-diagenesis in salt marsh sediments from the Medway Estuary, Kent, UK. Estuar Coast Shelf Sci 57: 43–54.

[21] Weis JS, Weis P (2004) Metal uptake, transport and release by wetland plants: implications for phytoremediation and restoration. Environ Int 30: 685–700. 15051245 10.1016/j.envint.2003.11.002

[22] Zwolsman JJG, Berger GW, Vaneck GTM (1993) Sediment accumulation rates, historical input, postdepositional mobility and retention of major elements and trace-metals in salt-marsh sediments of the scheldt estuary, SW Netherlands. Mar Chem 44: 73–94.

[23] Meire P, Ysebaert T, Damme SV, Bergh EVd, Maris T, et al (2005) The Scheldt estuary: a description of a changing ecosystem. Hydrobiol 540: 1–11.

[24] Baeyens W, Monteny F, Van Ryssen R, Leermakers M (1997) A box-model of metal flows through the Scheldt estuary (1981–1983 and 1992–1995). Hydrobiol 366: 109–128.

[25] Du Laing G, Vandecasteele B, De Grauwe P, Moors W, Lesage E, et al (2007) Factors affecting metal concentrations in the upper sediment layer of intertidal reedbeds along the river Scheldt. J Environ Monit 9: 449–455. 17492090 10.1039/b618772b

[26] Teuchies J, Beauchard O, Jacobs S, Meire P (2012) Evolution of sediment metal concentrations in a tidal marsh restoration project. Sci Total Environ 419: 187–195. 22297250 10.1016/j.scitotenv.2012.01.016

[27] Vandecasteele B, De Vos B, Tack FMG (2003) Temporal-spatial trends in heavy metal contents in sediment-derived soils along the Sea Scheldt river (Belgium). Environ Pollut 122: 7–18. 12535591 10.1016/s0269-7491(02)00282-8

[28] Beauchard O, Jacobs S, Cox TJS, Maris T, Vrebos D, et al (2011) A new technique for tidal habitat restoration: Evaluation of its hydrological potentials. Ecol Engin 37: 1849–1858.

[29] Cox T, Maris T, De Vleeschauwer P, De Mulder T, Soetaert K, et al (2006) Flood control areas as an opportunity to restore estuarine habitat. Ecol Engin 28: 55–63.

[30] Maris T, Cox T, Temmerman S, De Vleeschauwer P, Van Damme S, et al (2007) Tuning the tide: creating ecological conditions for tidal marsh development in a flood control area. Hydrobiol 588: 31–43.

[31] Struyf E, Temmerman S, Meire P (2007) Dynamics of biogenic Si in freshwater tidal marshes: Si regeneration and retention in marsh sediments (Scheldt estuary). Biogeochemistry 82: 41–53.

[32] Temmerman S, Govers G, Meire P, Wartel S (2003) Modelling long-term tidal marsh growth under changing tidal conditions and suspended sediment concentrations, Scheldt estuary, Belgium. Marine Geology 193: 151–169.

[33] Verlaan PAJ (2000) Marine vs Fluvial Bottom Mud in the Scheldt Estuary. Estuar Coast Shelf Sci 50: 627–638.

[34] Van Alsenoy V, Bernard P, Van Grieken R (1993) Elemental concentrations and heavy metal pollution in sediments and suspended matter from the Belgian North Sea and the Scheldt estuary. Sci Total Environ 133: 153–181.

[35] Zwolsman J (1999) Geochemistry of trace elements in the Scheldt estuary (Doctoral thesis). Utrecht. 183 p.

[36] Verlaan PAJ, Donze M, Kuik P (1998) MarinevsFluvial Suspended Matter in the Scheldt Estuary. Estuar Coast Shelf Sci 46: 873–883.

[37] Beeftink WG, Nieuwenhuize J, Stoeppler M, Mohl C (1982) Heavy-metal accumulation in salt marshes from the Western and Eastern Scheldt. Sci Total Environ 25: 199–223.

[38] Baeyens W, Leermakers M, Gieter MD, Nguyen HL, Parmentier K, et al (2005) Overview of trace metal contamination in the Scheldt estuary and effect of regulatory measures. Hydrobiol 540: 141–154.

[39] Van Damme S, Struyf E, Maris T, Ysebaert T, Dehairs F, et al (2005) Spatial and temporal patterns of water quality along the estuarine salinity gradient of the Scheldt estuary (Belgium and The Netherlands): results of an integrated monitoring approach. Hydrobiol 540: 29–45.

[40] Temmerman S, Moonen P, Schoelynck J, Govers G, Bouma TJ (2012) Impact of vegetation die-off on spatial flow patterns over a tidal marsh. Geophys Res Lett 39.

[41] Luoma SN, Rainbow PS, editors (2008) Metal Contamination in Aquatic Environments. Science and Lateral Management. first ed. New Yor: Cambridge University Press. 573 p.

[42] Masson M, Schäfer J, Blanc G, Dabrin A, Castelle S, et al (2009) Behavior of arsenic and antimony in the surface freshwater reaches of a highly turbid estuary, the Gironde Estuary, France. Appl Geochem 24: 1747–1756.

[43] Buggy CJ, Tobin JM (2008) Seasonal and spatial distribution of metals in surface sediment of an urban estuary. Environ Pollut 155: 308–319. 18207295 10.1016/j.envpol.2007.11.032

[44] Fettweis M, Sas M, Monbaliu J (1998) Seasonal, Neap-spring and Tidal Variation of Cohesive Sediment Concentration in the Scheldt Estuary, Belgium. Estuar Coast Shelf Sci 47: 21–36.

[45] Temmerman S, Govers G, Wartel S, Meire P (2003) Spatial and temporal factors controlling short-term sedimentation in a salt and freshwater tidal marsh, Scheldt estuary, Belgium, SW Netherlands. Earth Surface Processes and Landforms 28: 739–755.

[46] Chaudry M, Zwolsman J (2008) Seasonal Dynamics of Dissolved Trace Metals in the Scheldt Estuary: Relationship with Redox Conditions and Phytoplankton Activity. Estuaries and Coasts 31: 430–443.

[47] Paucot H, Wollast R (1997) Transport and transformation of trace metals in the scheldt estuary. Mar Chem 58: 229–244.

[48] Gerringa LJA, de Baar HJW, Nolting RF, Paucot H (2001) The influence of salinity on the solubility of Zn and Cd sulphides in the Scheldt estuary. J Sea Res 46: 201–211.

[49] Baeyens W, Brauwere A, Brion N, Gieter MD, Leermakers M (2007) Arsenic speciation in the River Zenne, Belgium. Sci Total Environ 384: 409–419. 17619054 10.1016/j.scitotenv.2007.05.044

[50] Baeyens W, van Eck B, Lambert C, Wollast R, Goeyens L (1997) General description of the Scheldt estuary. Hydrobiol 366: 1–14.

[51] Rozan TF, Benoit G (1999) Heavy metal removal efficiencies in a river-marsh system estimated from patterns of metal accumulation in sediments. Mar Environ Res 48: 335–351.

[52] Wartel S (1977) Composition, transport and origin of sediments in the Schelde estuary. Geol Mijnbouw 56: 219–233.

[53] Claus J, Ides S, De Mulder T, Mostaert F (2009) Baggeren en storten in de Schelde – Onderzoek naar de slibhuishouding in de Zeeschelde. WL Rapporten, 770_42. Antwerpen, België Waterbouwkundig Laboratorium & Universiteit Gent.

[54] Sas M, De Jonghe E (1993) Behaviour of particulate material in the Scheldt estuary. Final report for the MUMM (Ministry of Public Health, Belgium) (in Dutch).

[55] Van der Kooij LA (1982) The waterqualility of the Westerschelde in the period 1964–1981 (in Dutch). Rijksinstituut voor Zuivering van Afvalwater, Hoofdafdeling oppervlaktewater.

[56] Van Eck GTM, De Pauw N, Van Den Langenbergh M, Verreet G (1991) Emissions, concentrations, behaviour and effects of microcontaminants in the catchment of the Scheldt and Scheldt estuary (in Dutch). Water 60: 164–181.

[57] Flemish Government (2007) Westerschelde and Zeeschelde, dredging 2007 (in Dutch). Vlaamse overheid. Departement mobiliteit en Openbare Werken. Afdeling maritieme toegang.

[58] Walling DE, Owens PN (2003) The role of overbank floodplain sedimentation in catchment contaminant budgets. Hydrobiol 494: 83–91.

[59] Rozan TF, Benoit G (2001) Mass balance of heavy metals in New Haven Harbor, Connecticut: Predominance of nonpoint sources. Limnol Oceanogr 46: 2032–2049.

[60] Birch G (2011) Contaminated soil and sediments in a highly developed catchment-estuary system (Sydney estuary, Australia): an innovative stormwater remediation strategy. J Soils Sediments 11: 194–208.

[61] Overesch M, Rinklebe J, Broll G, Neue HU (2007) Metals and arsenic in soils and corresponding vegetation at Central Elbe river floodplains (Germany). Environ Pollut 145: 800–812. 16996182 10.1016/j.envpol.2006.05.016

[62] Pan K, Wang WX (2012) Trace metal contamination in estuarine and coastal environments in China. Sci Total Environ 421: 3–16. 21470665 10.1016/j.scitotenv.2011.03.013

[63] Pope N, Langston W (2011) Sources, distribution and temporal variability of trace metals in the Thames Estuary. Hydrobiol 672: 49–68.

[64] Weis JS, Bergey L, Reichmuth J, Candelmo A (2011) Living in a Contaminated Estuary: Behavioral Changes and Ecological Consequences for Five Species. Bioscience 61: 375–385.

