# Supplementary material for: Estuaries as Filters: The Role of Tidal Marshes in Trace Metal Removal
Source: PLoS One. 2013 Aug 7;8(8):e70381. doi: 10.1371/journal.pone.0070381 (PMC3737196; doi:10.1371/journal.pone.0070381)
Supplement: Table S1 — Overview of the data used for the calculation of the sediment deposition on the marshes in the different zones of the estuary. (DOCX) [file pone.0070381.s001.docx]

**Supplementary material**

SI. Table 1. Overview of the data used for the calculation of the sediment deposition on the marshes in the different zones of the estuary. See chapter 2.4.2. of the material and method section for detailed description.
