# Supplementary material for: Estuaries as Filters: The Role of Tidal Marshes in Trace Metal Removal
Source: PLoS One. 2013 Aug 7;8(8):e70381. doi: 10.1371/journal.pone.0070381 (PMC3737196; doi:10.1371/journal.pone.0070381)
Supplement: Table S2 — Metal concentrations in sediments (from cores or sediment traps) used for the calculation of metal deposition in the Schelde estuary. (DOCX) [file pone.0070381.s002.docx]

**Supplementary material**

SI. Table 2. Metal concentrations in sediments (from cores or sediment traps) used for the calculation of metal deposition in the Schelde estuary. INBO = *Flemish (Belgium) governmental research institute for nature and forest*. RWS = *the Dutch ministry for infrastructure and environment, Rijkswaterstaat*.
